# Paving the way for methane hydrate formation on metal–organic frameworks (MOFs)†
†Electronic supplementary information (ESI) available: Water adsorption isotherms, textural characterization analysis of the different MOFs before and after pre-humidification, XRD analysis before and after the methane hydrate formation process, and adsorption kinetic measurements. See DOI: 10.1039/c6sc00272b


**DOI:** 10.1039/c6sc00272b

**Published:** 2016-02-19

**Authors:** Mirian E. Casco, Fernando Rey, José L. Jordá, Svemir Rudić, François Fauth, Manuel Martínez-Escandell, Francisco Rodríguez-Reinoso, Enrique V. Ramos-Fernández, Joaquín Silvestre-Albero

**Affiliations:** a Laboratorio de Materiales Avanzados , Departamento de Química Inorgánica-Instituto Universitario de Materiales , Universidad de Alicante , Ctra. San Vicente-Alicante s/n , E-03690 San Vicente del Raspeig , Spain . Email: joaquin.silvestre@ua.es; b Instituto de Tecnología Química , Universidad Politécnica de Valencia-CSIC , Avda. de los Naranjos, s/n , E-46022 Valencia , Spain; c ISIS Facility , Rutherford Appleton Laboratory , Chilton , Didcot , UK OX11 0QX; d ALBA Light Source , E-08290 Cerdanyola del Vallés , Barcelona , Spain

## Abstract

The presence of a highly tunable porous structure and surface chemistry makes metal–organic framework (MOF) materials excellent candidates for artificial methane hydrate formation under mild temperature and pressure conditions (2 °C and 3–5 MPa). Experimental results using MOFs with a different pore structure and chemical nature (MIL-100 (Fe) and ZIF-8) clearly show that the water–framework interactions play a crucial role in defining the extent and nature of the gas hydrates formed. Whereas the hydrophobic MOF promotes methane hydrate formation with a high yield, the hydrophilic one does not. The formation of these methane hydrates on MOFs has been identified *for the first time* using inelastic neutron scattering (INS) and synchrotron X-ray powder diffraction (SXRPD). The results described in this work pave the way towards the design of new MOF structures able to promote artificial methane hydrate formation upon request (confined or non-confined) and under milder conditions than in nature.

## Abstract 2

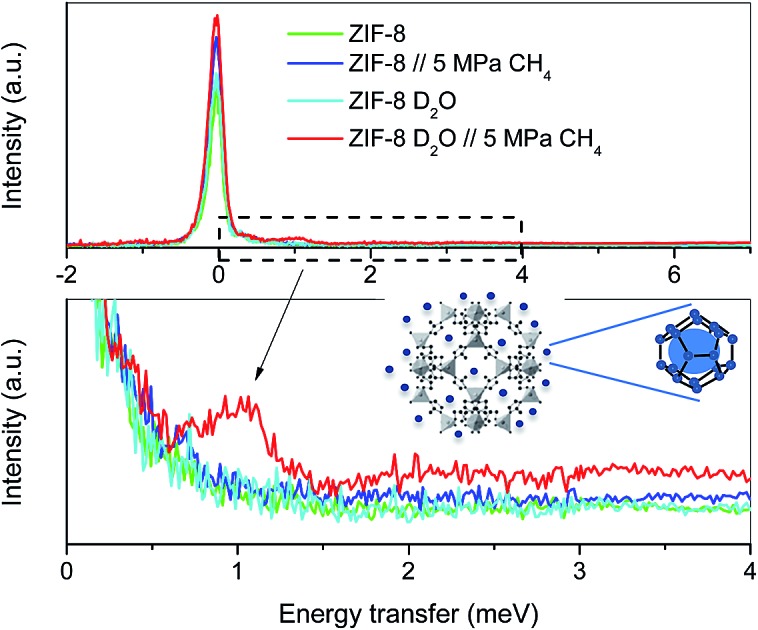
The formation of methane hydrates on MOFs has been identified for the first time using inelastic neutron scattering and synchrotron X-ray powder diffraction.

## Introduction

The large depletion of fossil fuels anticipated in the past decades has shifted the attention of governments and expert panels towards new fuel sources, mainly shale gas and methane hydrates. These two natural sources constitute the most promising reservoirs for light hydrocarbons on Earth able to fulfil the energetic requirements of modern society for the next decades. Due to their relevance for the worldwide economy, urgent research is required (i) to find new storage/transportation technologies (*e.g.*, adsorption in nanoporous solids) for these light hydrocarbons (mainly methane) for their use in mobile applications and (ii) to understand their growth/exploitation mechanism, in the specific case of methane hydrates.^1^,^2^

Natural methane hydrates are crystalline solids that form in nature when methane and water come into contact under thermodynamically favorable conditions, that is, high pressure (typically more than 6 MPa) and relatively low temperature (slightly below room temperature), giving rise to an ice-like hydrogen-bonded structure.^3^ These natural methane reservoirs are located in deep-sea sediments and the permafrost. Actual prospections have estimated that the amount of energy in the form of hydrates may be twice that of all other fossil fuels combined.^2^ Since the first onshore production tests at the Mallik site (Canada) in 2002, several industrial projects have been performed around the world (*e.g.*, MH21 research consortium in Japan), with the aim of recovering natural gas from deep-under-sea natural methane hydrate reservoirs using preferentially two approaches: thermal stimulation (*e.g.*, pumping hot water) or depressurization.^3^ However, there are still many open questions and technological issues that must be understood (*e.g.*, methane hydrate formation/dissociation mechanism in confined space, thermal stability of methane hydrates, *etc.*) before the process can be properly commercialized (whereas the United States has no urgent need to mine methane hydrates, Japan plans to start its commercialization by the year 2018).^3^

Besides being a natural resource, methane hydrates can also be considered as a potential technology for natural gas storage and transportation provided that they can be artificially synthesized under mild temperature and pressure conditions, and within a reasonable timescale (the theoretical storage capacity of methane hydrates would be up to 180 volumes of natural gas per volume of hydrate).^4^ Methane, the main component of natural gas hydrates, exhibits important advantages as a fuel compared to gasoline and diesel in terms of energy density, energy efficiency and environmental concerns. Whereas storage of methane at low temperature (liquefied natural gas—LNG—at –162 °C) or at extremely high pressure (compressed natural gas—CNG—at 25 MPa) is highly undesirable from safety and energy-saving points of view, the use of confinement effects, *e.g.*, adsorption using nanoporous solids, the so-called adsorbed natural gas—ANG, has become a promising alternative to store methane at moderate temperatures and pressures. Among these nanoporous materials, metal–organic frameworks (MOFs) (mainly HKUST-1 and NiMOF-74) have been postulated as the best candidates to reach the new US Department of Energy (DOE) objective defined as 263 cm^3^ cm^–3^ or 0.5 g per g, at a moderate methane pressure, *ca.* 6–7 MPa.^5^,^6^ Besides MOFs, specially designed activated carbons containing a highly developed porous structure and a large BET surface area have also been postulated in the literature as promising materials to reach this target, although at a slightly higher pressure, *ca.* 10 MPa.^7^ However, further improvements are required to reach the new DOE target at a lower pressure, *ca.* 3–4 MPa, thus facilitating the use of these systems in domestic applications with simple one-stage compressors.

A step further in methane storage requires mimicking nature, *i.e.* to take advantage of the confinement effects inside the cavities of nanoporous materials, similar to deep-under-sea sediments, and to use them not only as physisorption media, as classically, but also as nanoreactors to nucleate and grow artificial methane hydrates. Indeed, recent studies from our research group have anticipated that properly designed activated carbons can be used as a guest structure to grow artificial methane hydrates under mild conditions (3.5 MPa and 2 °C), with faster kinetics than nature (within minutes), fully reversibly and with a nominal stoichiometry that mimics nature.^8^ The promotion of methane hydrate formation (nucleation and growth) has also been observed in porous silicas, silica sand and natural sediments.^9^–^12^ Despite these promising results, activated carbons^13^ and silica-based materials exhibit an important limitation associated with the lack of structural versatility, in terms of composition and/or surface functionality. Taking into account that metal–organic framework materials (MOFs) are porous systems combining a highly developed porous structure, a large surface area and a tunable porosity, surface chemistry and composition,^14^,^15^ these materials can *a priori* be envisaged as promising candidates to this end. Indeed, recent studies from Kim *et al.* have anticipated that MIL-53 can promote methane hydrate formation, the nucleation taking place exclusively in the interparticle space due to the small cavity in MIL-53 (∼0.6 nm) compared to the hydrate sI unit cell ∼1.2 nm.^16^

With this in mind, the aim of this study is to pave the way for artificial methane hydrate formation using metal–organic frameworks with pore cavities large enough to allocate methane hydrate nucleation and growth. A couple of MOFs, the hydrophilic MIL-100 (Fe) and hydrophobic ZIF-8, have been selected in order to evaluate the effect of the surface chemistry, porosity and amount of water in the methane hydrate nucleation process. Adsorption experiments in static conditions have been combined with inelastic neutron scattering (INS) experiments and synchrotron X-ray powder diffraction (SXRPD) measurements to prove *for the first time* that properly designed MOFs can be used as nanoreactors to grow artificial methane hydrates with the sI structure, thus improving the storage and working capacity of the parent MOF.

## Results and discussion

### High-pressure methane adsorption isotherms

As described above, the selection of the two MOF materials was not arbitrary and was based on their different porous structures and surface chemistry. MIL-100 (Fe) is a hydrophilic material (water adsorption capacity at 25 °C and *p*/*p*_0_ ≈ 0.95 is *ca.* 0.56 g per g, see Fig. S1†), with large mesoporous cavities, *ca.* 2.4–2.9 nm, accessible *via* 0.55 nm and 0.86 nm windows. The N_2_ adsorption/desorption isotherm for MIL-100 (Fe) synthesized using a microwave-assisted solvothermal route exhibits a narrow knee at low relative pressures characteristic of a microporous material (Fig. S2†). The synthesized sample exhibits a BET surface area of 1476 m^2^ g^–1^ and a total micropore volume of 0.87 cm^3^ g^–1^, in close agreement with previous results described in the literature.^17^ On the other hand, commercial ZIF-8 exhibits a highly hydrophobic surface (water adsorption capacity at 25 °C and *p*/*p*_0_ ≈ 0.95 is *ca.* 0.018 g per g, Fig. S1†), with inner cavities around 1.2 nm, accessible *via* 6-ring windows of *ca.* 0.44 nm. ZIF-8 exhibits a type I nitrogen adsorption isotherm with characteristic steps at *p*/*p*_0_ ≈ 0.70 kPa and 2.5 kPa, in close agreement with the literature.^18^ The BET surface area of ZIF-8 is 1565 m^2^ g^–1^, with a micropore volume of 0.72 cm^3^ g^–1^.

**Fig. 1:**
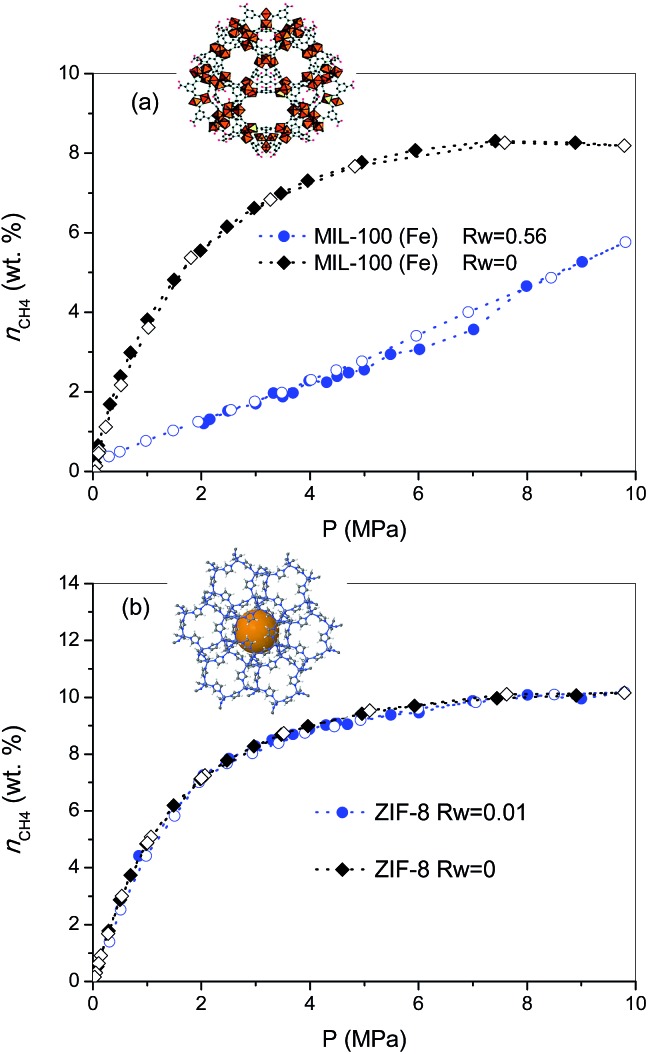
Methane adsorption (full symbols)/desorption (empty symbols) isotherms at 2 °C and up to 10 MPa for samples (a) MIL-100 (Fe) and (b) ZIF-8, in the absence (*R*_w_ = 0) and in the presence of humidity (*R*_w_ = 0.56 g per g, for MIL-100 (Fe), and *R*_w_ = 0.01 g per g, for ZIF-8) (wt% = g_CH_4__/100 g_dry carbon_).

The excess methane adsorption/desorption isotherms for the different MOFs selected were measured in dry and in pre-humidified (saturated) samples at 2 °C and up to 10 MPa. As can be observed from Fig. 1, the dry forms of MIL-100 (Fe) and ZIF-8 samples exhibit a type I isotherm, according to the IUPAC classification, with a progressive increase in the amount adsorbed up to a plateau at 8–9 MPa.^19^ The total excess amount adsorbed at 2 °C reaches a value as high as 8.3 wt%, for MIL-100 (Fe), and 10.2 wt%, for the case of ZIF-8. Interestingly, both adsorption isotherms are fully reversible over the whole pressure range evaluated, thus suggesting the absence of strong adsorbate–adsorbent interactions. Although these values are quite promising among inorganic solids, they are still far from those obtained using petroleum-pitch derived activated carbons (25.5 wt%) or similar MOF materials such as HKUST-1 (21.1 wt%), at a similar pressure but at a slightly higher temperature (25 °C *vs.* 2 °C).^7^ As described above, whereas MIL-100 (Fe) is based on trimesic acid as linker containing three carboxylic groups, ZIF-8 is based on 2-methylimidazole as linker, *i.e.* MIL-100 (Fe) is a hydrophilic material (due to the presence of coordinatively unsaturated sites), whereas ZIF-8 is hydrophobic. Furthermore, MIL-100 (Fe) exhibits large cavities (*ca.* 2.4–2.9 nm) able to accommodate up to two unit cells of methane hydrate, whereas cavities in ZIF-8 are *ca.* 1.2 nm, the size of the unit cell for methane hydrate with a sI structure.^4^ Upon saturation with 90% relative humidity at 25 °C (saturation achieved is 0.56 g H_2_O per g_dry MOF_ for MIL-100 (Fe), and 0.01 g H_2_O per g_dry MOF_ for ZIF-8), both samples were evaluated in the adsorption of methane at 2 °C and up to 10 MPa. As observed from Fig. 1, the adsorption behaviour of the pre-humidified samples highly differs depending on the MOF evaluated. In the case of a hydrophilic sample such as MIL-100 (Fe) the methane adsorption isotherm exhibits a drastic decrease in the amount adsorbed as compared to the dry sample over the whole pressure range evaluated, the final amount adsorbed at 10 MPa reaching a value of 5.8 wt%. The sudden decrease observed in the methane adsorption capacity of MIL-100 (Fe) upon moisture exposure clearly demonstrates the blockage of the porosity by pre-adsorbed water. Although the methane adsorption isotherm in the wet sample is fully reversible, a closer look to the mid-high pressure region (∼5–8 MPa) denotes a slight deviation between the adsorption and the desorption branches. The presence of a small hysteresis loop in this pressure region and a certain step in the amount adsorbed at 7 MPa, are clear fingerprints for the methane hydrate nucleation in the inner cavities of the MIL-100 (Fe). The high pressure threshold (7 MPa) for methane hydrate nucleation in the narrow cavities of MIL-100 (Fe) is in close agreement with previous measurements on petroleum-pitch derived carbon materials (PP-AC), although with an extremely low yield in the case of MOFs (only a small amount of the water pre-adsorbed is converted to methane hydrate).^8^ The low extent of methane hydrate nucleation and growth in pre-humidified MIL-100 (Fe) as compared to hydrophobic carbon clearly anticipates that, despite having large cavities (2.4–2.9 nm) and a high BET surface area, the presence of strong water–framework interactions does not promote methane hydrate formation in the confined space. Apparently, small water–adsorbent interactions are required to promote the preferential water–methane interactions needed for the nucleation and growth of methane hydrates.

To further explore this assumption, the pre-humidification step has been applied to a hydrophobic MOF such as ZIF-8 (saturation close to 0.01 g_H_2_O_ per g_dry ZIF-8_). As can be observed from Fig. 1b, the excess methane adsorption isotherm for the saturated sample perfectly fits the profile for the dry material. Apparently, the highly hydrophobic nature of ZIF-8 limits the extent of water pre-adsorbed, thus excluding any possibility for methane hydrate formation. Interestingly, saturated ZIF-8 keeps the whole porosity fully available for the adsorption of methane molecules, *i.e.*, there are neither blocking effects nor remaining water in the pore mouth. The results obtained for the saturated samples are in close agreement with their N_2_ adsorption isotherms (Fig. S3†), showing that whereas saturated MIL-100 (Fe) exhibits a drastic decrease in the nitrogen adsorption capacity, associated with the pore blocking by water present inside the hydrophilic cavities, the porous structure of ZIF-8 remains unaltered, thus confirming that water is completely rejected from the inner hydrophobic cavities.

**Fig. 2:**
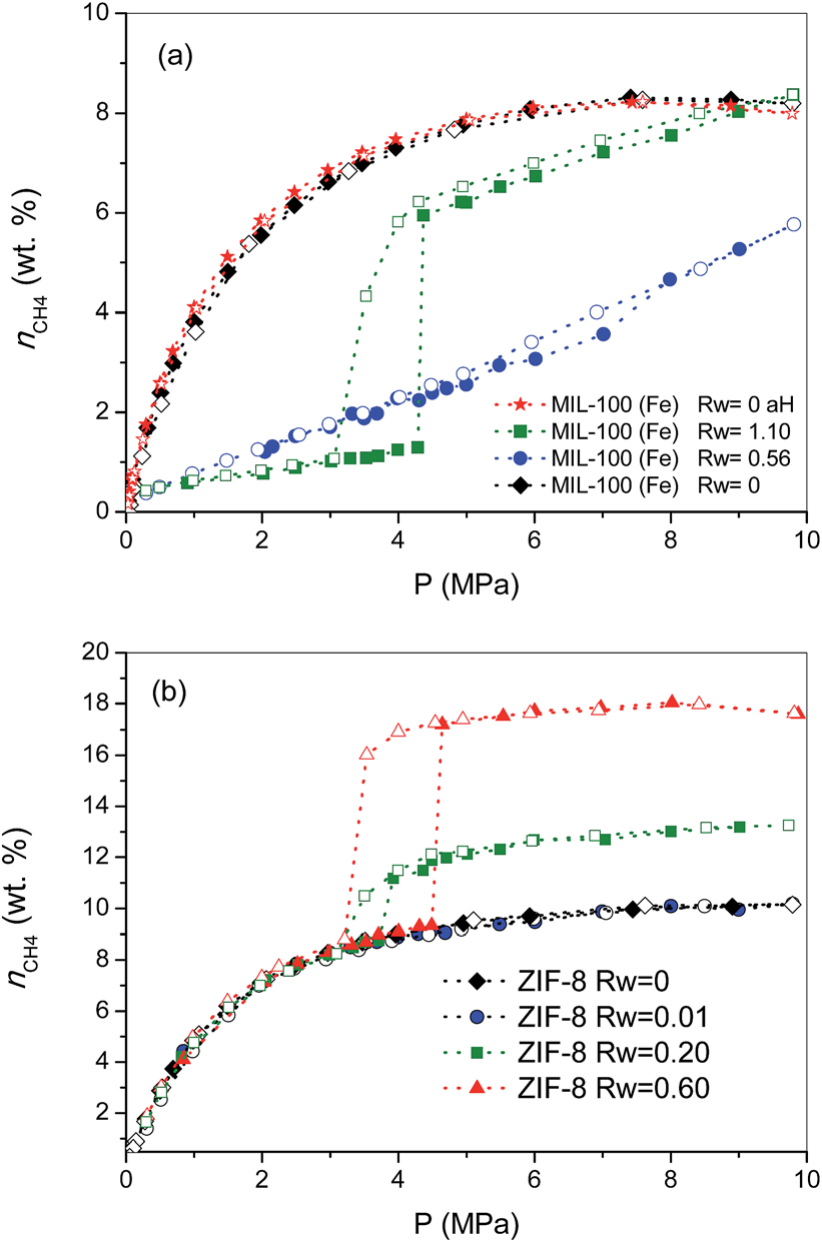
Effect of pre-humidification conditions in the methane adsorption (full symbols)/desorption (empty symbols) isotherms for samples (a) MIL-100 (Fe) and (b) ZIF-8 at 2 °C and up to 10 MPa. Dried MIL-100 (Fe) regenerated after the hydrate formation process (aH) has been included for the sake of comparison (wt% = g_CH_4__/100 g_dry carbon_).

To gain a deeper knowledge about the effect of the pre-humidification conditions, the adsorption experiments were extended to oversaturated samples. The oversaturated samples were prepared by additional incorporation of water droplets with a syringe up to *R*_w_ = 1.10 g H_2_O per g_dry MOF_, for MIL-100 (Fe), and up to *R*_w_ = 0.2 and 0.6 g H_2_O per g_dry MOF_, for ZIF-8. Fig. 2 shows the excess methane adsorption isotherms up to 10 MPa for oversaturated (a) MIL-100 (Fe) and (b) ZIF-8 at 2 °C. Oversaturated MIL-100 (Fe) exhibits a similar behaviour to the saturated sample in the low-pressure region. The presence of water inside the cavities highly inhibits methane uptake up to *ca.* 4.3 MPa. Above this threshold pressure, there is a sudden jump in the amount of methane adsorbed up to 6 wt%, the methane adsorption isotherm following a similar profile to the saturated sample thereafter. The drastic increase in the amount adsorbed and the associated hysteresis loop in the mid-pressure window (3–4.5 MPa) clearly anticipate the methane hydrate formation in MIL-100 (Fe). However, the final amount adsorbed at 10 MPa (8.4 wt%) does not improve the adsorption capacity for the dry material, in contrast to previous measurements using activated carbon materials.^8^ The low adsorption capacity of the oversaturated MIL-100 (Fe) compared to carbon materials, under similar pre-humidification conditions, must be associated with the low water-to-hydrate yield. According to the water adsorption isotherms (Fig. S1†), the amount of water accommodated at saturation in the inner cavities of MIL-100 (Fe) is 0.56 g H_2_O per g. Consequently, the additional 0.54 g H_2_O per g in the oversaturated sample (up to 1.10 g H_2_O per g) must be allocated in the external surface and/or in the interparticle space. Taking into account the blocking effects of water present in the inner cavities for the saturated MIL-100 (Fe) and the presence of the threshold pressure at around 4 MPa (characteristic of methane hydrate formation in large cavities),^8^ one can assume that methane hydrate formation will take place, preferentially, in the external surface of the MOF. Under this assumption, the surface water-to-adsorbed methane ratio in the region of the jump gives a value as high as 10.20, far above the theoretical stoichiometric value of 5.75 (1CH_4_·5.75H_2_O). This observation suggests that only 56% of the water in the external surface participates in the hydrate formation process. This finding is quite understandable taking into account that even in the outer surface water will experience strong interactions with the MIL-100 (Fe) framework, with the corresponding inhibition in the conversion of water-to-hydrate, even at high pressures. Lastly, the oversaturated sample was evaluated in the adsorption of methane in a second cycle after an outgassing treatment at 110 °C for 12 h to remove the pre-adsorbed water. As can be observed from Fig. 2a, the methane adsorption isotherm of the regenerated sample (after the hydrate formation—aH) fully overlaps with the original one, thus reflecting that neither the methane hydrate formation nor the water pre-humidification step produce any damage and/or deterioration in the porous network of the MIL-100 (Fe). This observation has been further confirmed by XRD analysis performed before and after these experiments (see Fig. S4†).

Concerning the oversaturated ZIF-8 samples, the scenario changes completely compared to MIL-100 (Fe). Fig. 2b shows the excess methane adsorption isotherms for samples oversaturated with 0.2 g H_2_O per g and 0.6 g H_2_O per g. As can be observed, the adsorption isotherm for the sample with *R*_w_ = 0.2 g per g perfectly fits the profile for the dry material up to *ca.* 3.7 MPa. Surprisingly, there is a sudden jump in the adsorption isotherm above this pressure threshold, *ca.* 2.2 wt% CH_4_ increase, which remains mainly constant with pressure up to 10 MPa. The methane isotherm is fully reversible over the whole pressure range evaluated; no hysteresis loop can be observed, except in the region of the step where a small deviation between the adsorption and desorption branch can be appreciated. An increase in the amount of water incorporated (*R*_w_ up to 0.6 g per g) gives rise to (i) a further increase in the magnitude of the jump (*ca.* 8.0 wt%), (ii) no interference in the low pressure region, (iii) a shift of the jump to higher pressures (around 4.5 MPa) and (iv) the appearance of a remarkable hysteresis loop. A closer look at the isotherm shows that the larger hysteresis loop in the sample with 0.6 g per g must be attributed to the shift in the adsorption branch to higher pressures, since the desorption branch is fully coincident independently of the *R*_w_ (desorption cycles always close at 3.2 MPa). In other words, the nucleation process may be metastable on larger water droplets, whereas the methane hydrate decomposition must be crystal-size independent. The crystallinity of the used samples after the methane hydrate formation process (Fig. S4†) excludes any structural damage after these processes.

Previous studies from our research group using activated carbons anticipated that methane hydrate formation in large pores (wide mesopores and macropores) takes place in the mid-pressure region (around 3–4 MPa), whereas larger pressures (above 6 MPa) are required for methane hydrate formation in small cavities, at least when diffusional restrictions are expected.^8^ Taking into account these premises, the results observed for ZIF-8 suggest some important findings: (i) the perfect fitting in the amount adsorbed up to 3–4.5 MPa anticipates that the porosity in ZIF-8 remains fully available after the pre-humidification step, independently of the amount of water incorporated; (ii) the high hydrophobicity of the ZIF-8 surface seems to inhibit moisture to access the inner porosity, so that small water nanodroplets must be formed in the external surface of the MOF and/or in the interparticle space; (iii) the jump observed in the methane adsorption isotherm in the mid-pressure region must be associated with methane hydrate formation in large cavities (maybe in the interparticle space), or in the external surface; and (iv) the quasi-vertical jump in the isotherm clearly suggests the formation of highly homogeneous methane hydrate nanocrystals, most probably in the aforementioned water nanodroplets.

To end, the amount of methane adsorbed in the step at medium pressure was correlated with the amount of water incorporated, assuming that all water participates in the hydrate formation process, to determine the stoichiometry of the synthesized hydrates. The values calculated are 1CH_4_·5.75H_2_O, for the sample oversaturated with *R*_w_ = 0.2, and 1CH_4_·5.9H_2_O, for the oversaturated ZIF-8 samples with *R*_w_ = 0.6. These results show that in the case of hydrophobic surfaces, water is prone to form methane hydrate nanocrystals with a water-to-methane hydrate yield close to 100% and with a stoichiometry that mimics natural hydrates (1CH_4_·5.75H_2_O).^4^ The high water-to-hydrate yield is in close correlation with previous results described in the literature for activated carbons, and slightly above the value for nanosilica suspensions (80–90%).^8^,^12^

**Fig. 3:**
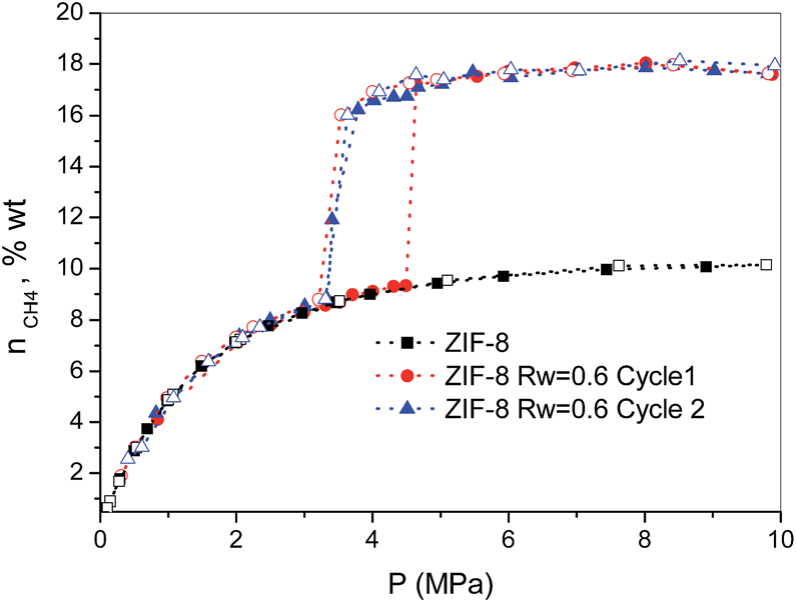
Methane adsorption (full symbols)/desorption (empty symbols) isotherms in pre-humidified ZIF-8 (*R*_w_ = 0.6) after different cycles (wt% = g_CH_4__/100 g_dry carbon_).

Finally, the methane hydrate formation in ZIF-8 was evaluated after successive cycles, *i.e.* once the desorption step from the first isotherm is finished the sample was re-evaluated without any additional thermal treatment or any further evacuation step. According to Fig. 3, whereas the first cycle is characterized by a shift in the pressure-threshold for methane hydrate formation to high pressures and the appearance of a hysteresis loop, the second cycle is fully reversible, *i.e.* there is a down-shift in the pressure-threshold in the second run (adsorption branch). This observation clearly reflects the well-known surface memory effect in gas hydrates, and it can be attributed to some preorganization of bulk water for hydrate formation after the first cycle (*e.g.*, retention of hydrogen-bonded 5-rings)^20^ or to the remaining methane dissolved in the water nanodroplets. Furthermore, the absence of a hysteresis loop in the second cycle suggests that the nucleation/decomposition of the methane hydrate nanocrystals takes place under full equilibrium conditions. In any case, the magnitude of the jump in the second cycle (*ca.* 7.9 wt%) perfectly fits with the first one, *i.e.* the methane hydrate nucleation and growth in ZIF-8 is highly recyclable with no detectable loss in the final storage capacity.

Another important parameter in the methane hydrate formation process concerns the nucleation kinetics. Fig. S5† shows the pressure changes with time in the reactor chamber for the first point in the isotherm right after the jump, *i.e.*, the point where the methane hydrate formation takes place, for the ZIF-8 sample pre-humidified with (a) *R*_w_ = 0.2 and (b) *R*_w_ = 0.6. When compared to activated carbon materials, the scenario changes completely in the case of MOFs. For both moisture ratios, after an initial rapid gas dissolution, there is an induction period that lasts between 2 and 4 h, depending on *R*_w_, before the methane hydrate growth process takes place. The induction period involves the initial clustering process to form partial hydrates and the formation of a critical size cluster.^21^,^22^ According to Fig. S5,† despite being in thermodynamically favourable conditions, the induction period highly depends on the amount of pre-adsorbed water, *i.e.* the size of the water nanodroplets. The presence of an induction period is in close agreement with the low solubility of methane in water and its low diffusion coefficient (*ca.* 0.7 × 10^–9^ m^2^ s^–1^ at 0 °C).^23^ However, previous studies described in the literature have shown that the induction time can be decreased with an increase in the water contact angle, *i.e.*, with an increase in the hydrophobicity of the solid surface.^24^ Furthermore, these studies have shown a decrease in the induction period for smaller water droplets for gas hydrate formation in hydrophobized sand particles. Apparently, water molecules in the vicinity of a hydrophobic surface are prone to nucleate partial hydrates due to the mismatch between both surfaces (through stabilization of 5–8 ring defects).^25^ Based on these premises, the larger liquid–solid interphase in small water nanodroplets present in ZIF-8 *R*_w_ = 0.2 could explain the shorter induction period in this sample. Once the critical crystal size is achieved (*ca.* 10–30 nm),^26^ after the induction period, the crystal growth zone starts giving rise to a sudden decrease in the manifold pressure. Fig. S6† shows that the kinetics for hydrate growth are slightly faster in the sample with *R*_w_ = 0.6, which can be attributed to the relatively higher pressure in the manifold or to the higher concentration of methane after a larger induction period. In any case, the growth of the hydrate crystals is relatively fast in both samples (less than 2 h to reach more than 90% methane entrapment).^27^

In summary, these results show that using ZIF-8 as a guest structure it is possible to design or model two step charge/discharge devices for methane storage with improved storage properties. Whereas the first adsorption process is constant and takes place in the inner porosity, the second adsorption process can be tailored to improve the adsorption performance (up to 85% improvement in the amount of methane adsorbed after incorporating 0.6 g_H_2_O_ per g) *via* nucleation and growth of methane hydrate nanocrystals in the external surface and/or in the interparticle space of the MOF. At this point it is important to highlight that similar experiments with methane and bulk water but in the absence of MOFs, do not provide any sign of methane adsorption, dissolution and/or nucleation (at least after more than two weeks), thus reflecting the critical role of the metal–organic frameworks (MOFs) in promoting the water–methane interactions. These findings open the door for the design of new MOF materials with tailored porous structure and surface chemistry to achieve proper methane hydrate nucleation and growth, either confined or non-confined, depending on the final application.

### Inelastic neutron scattering of methane hydrates

**Fig. 4:**
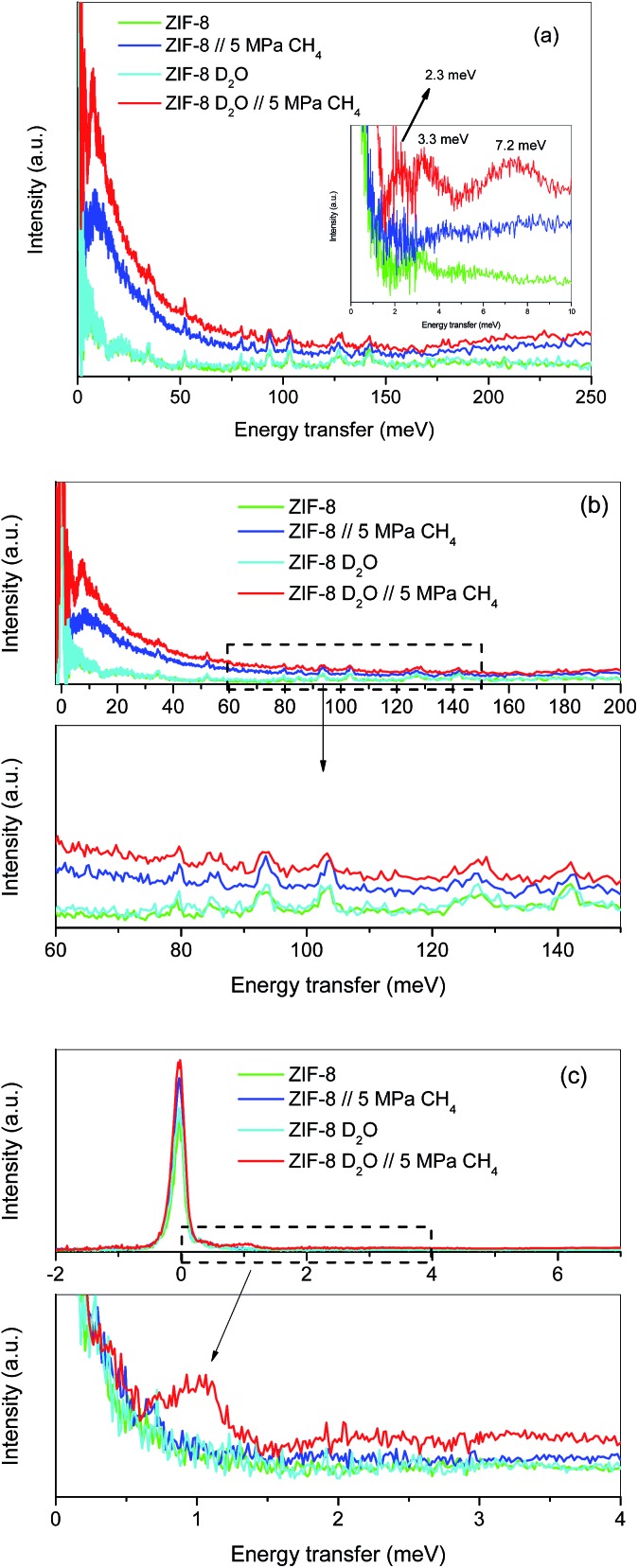
Inelastic neutron scattering spectra of ZIF-8 as-received and pre-impregnated with deuterated water (*R*_w_ = 0.7) before and after exposure to 5 MPa methane at 2 °C; (a) general overview, (b) high energy transfer region and (c) low-energy transfer region.

Although the methane adsorption isotherms described above have predicted the possible formation of methane hydrates on metal–organic framework materials, *in situ* high-resolution techniques are required to confirm their formation and to identify their structure. Among them, inelastic neutron scattering (INS) is a very useful technique based on the scattering of neutrons by atoms, the energy loss being associated with the atomic displacement (rotation and vibration) of the scattering atoms. In addition, an advantage of INS concerns the uniquely high neutron incoherent scattering cross-section of hydrogen, which is very interesting when evaluating organic scaffolds or molecules involving hydrogen (such as CH_4_). For a better evaluation of the MOF framework and the CH_4_ molecules, INS experiments were performed using D_2_O (0.7 g per g; 0.7 g D_2_O per g corresponds to 0.6 g H_2_O per g), instead of H_2_O, to reduce the parasitic scattering from the water framework. These experiments were performed using the TOSCA instrument at the ISIS Neutron and Muon Pulsed Source, Rutherford Appleton Laboratory in the United Kingdom. INS experiments were limited to ZIF-8 due to its better performance in terms of methane adsorption capacity compared to MIL-100 (Fe). Fig. 4a shows the inelastic neutron scattering (INS) spectra for ZIF-8 both in the dry (*R*_w_ = 0 g per g) and oversaturated (*R*_w_ = 0.7 g_D_2_O_ per g_dry ZIF-8_) forms, before and after the incorporation of 5 MPa of methane. The final pressure was selected in order to ensure the methane hydrate formation. The INS spectra were measured up to an energy transfer of 250 meV, in order to cover the most relevant rotational and vibrational modes of the zeolitic–imidazole framework, in addition to any contribution coming from the methane gas molecules incorporated. The spectra for the parent MOF, either dry or wet, are very similar among them, with the elastic contribution at 0 meV, and the appearance of additional peaks in the middle-energy region (75–150 meV), attributed to in-plane and out-of-plane deformations of the aromatic linker and C–C and C–N stretching modes.^28^–^30^ A closer look to the terahertz region (see inset for an amplification) allows a contribution around 3.1–3.2 meV to be discerned, attributed to the dynamics of the framework opening in ZIF-8.^29^,^30^

The incorporation of 5 MPa of methane in the dry MOF has no effect in the low energy region, except for the expected increase in the background signal due to the molecular recoil of methane that washes out any spectroscopic information (see inset). This behaviour is due to the light mass of methane and the presence of weak intermolecular interactions. A closer evaluation of the high-energy region (see Fig. 4b) shows a perfectly fitting profile with the original ZIF-8, *i.e.*, there is no appreciable shift in the different vibration and rotational modes of the framework organic linkers upon high-pressure methane exposure, thus ruling out any significant structural deformation as raised recently in the literature for ZIF-8 upon exposure to nitrogen at sub-atmospheric pressures.^18^

A completely different scenario takes place for the D_2_O saturated ZIF-8 upon exposure to 5 MPa of methane for 5 h. Besides the free rotational mode of the methyl group from the imidazolate linker at 3.2 meV (second rotational transition *J* = 0 → *J* = 2), introduction of methane gives rise to additional signals in the terahertz region. Indeed, there are two new inelastic contributions appearing at *ca.* 2.3 meV and 7.2 meV (see inset in Fig. 4a) that must be unambiguously attributed to the different rotational transitions of methane behaving as an almost free rotor in methane hydrates, in close agreement with the INS spectra obtained for natural methane hydrates from the Pacific sea-floor and with artificial methane hydrates confined in activated carbons.^8^,^31^

At this point it is interesting to highlight that methane hydrates exhibit a third contribution in the terahertz region at 3.3 meV,^8^,^31^ although in the specific case of ZIF-8 it is difficult to distinguish it due to the overlapping with the methyl group contribution from the imidazolate linker. The almost free rotation of methane is a clear indication that guest molecules are isolated in the hydrate cages, thus avoiding intermolecular interactions that would wash out the INS spectra. Final evidence about the methane hydrate formation comes from the transition from the rotational ground state (*J* = 0) of the methane, as a free rotor, to the first excitation state (*J* = 1), usually appearing around 1.31 meV. A closer look to the elastic contribution at 0 meV (Fig. 4c) clearly denotes a marked shoulder in the D_2_O pre-impregnated MOF upon methane exposure with maxima at 1.03 meV, close to the value achieved for artificial methane hydrates on activated carbon materials.^8^

### Synchrotron X-ray powder diffraction of methane hydrates

To further confirm the presence of methane hydrates and to identify their crystalline structure, D_2_O pre-impregnated ZIF-8 (*R*_w_ = 0.7) was evaluated using synchrotron X-ray powder diffraction (SXRPD) at the high-pressure/microdiffraction end station of the MSPD beamline at synchrotron ALBA (Barcelona, Spain).^32^ After the oversaturation of the ZIF-8 sample with deuterated water, the sample was placed in an *ad hoc* high-pressure capillary cell (fused silica capillary) mounted in a stainless-steel platform and connected to an on-line gas system.

**Fig. 5:**
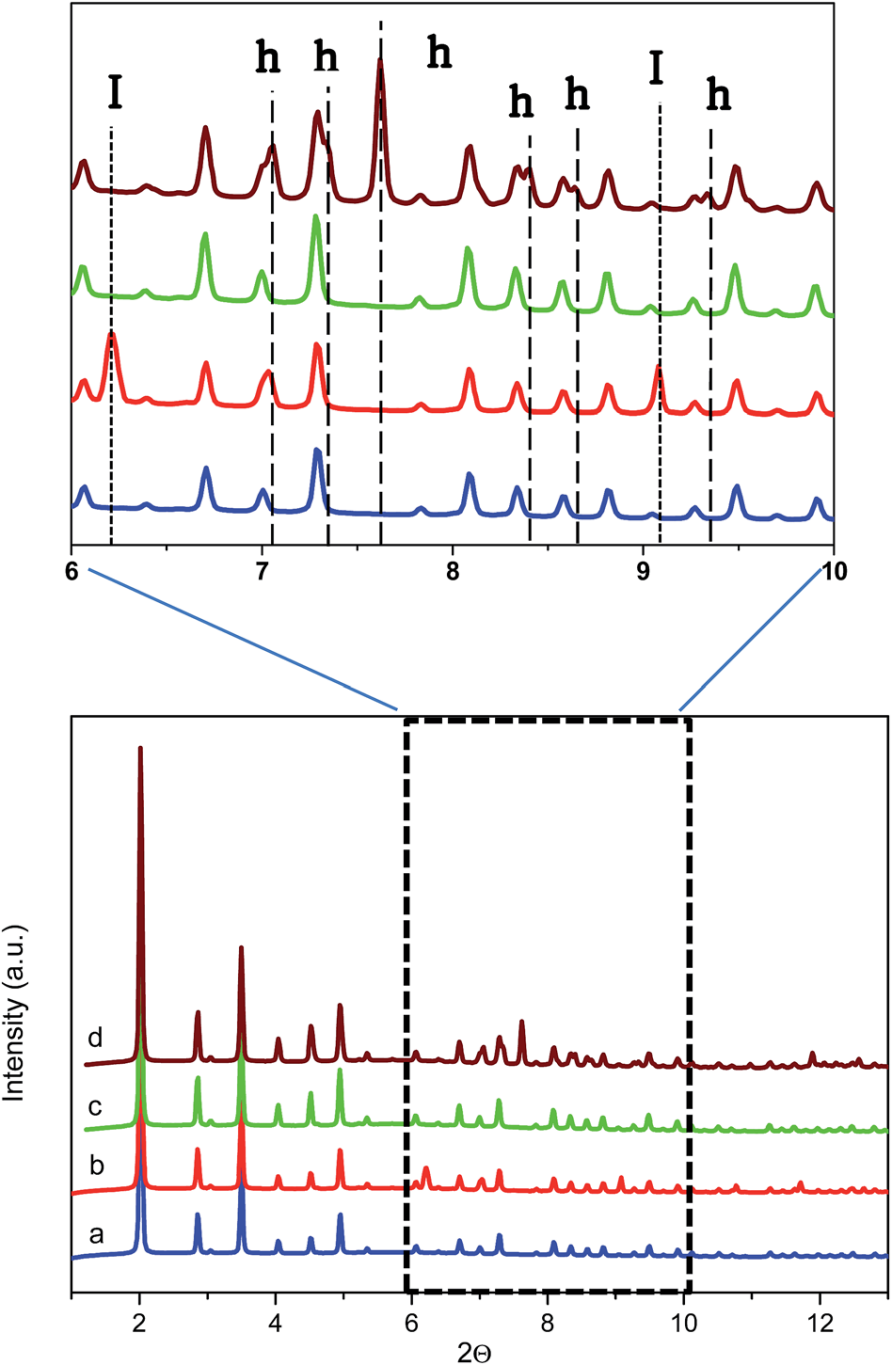
Synchrotron X-ray powder diffraction pattern of ZIF-8 oversaturated with D_2_O (a) at 5 °C in the absence of methane, (b) at –3 °C in the absence of methane, (c) at 5 °C in the presence of 5 MPa of methane and (d) at 2 °C in the presence of 5 MPa of methane (after 5 h induction period). Reflections corresponding to ice and hydrate are marked as I and h, respectively (*λ* = 0.4243 Å).

The SXRPD data of the wet sample at room temperature and in the absence of methane present the typical pattern corresponding to the ZIF-8 material (see Fig. 5a). A subsequent cooling step down to –3 °C gives rise to the appearance of diffraction peaks corresponding to the formation of ice with the hexagonal I_h_ phase (see dashed lines denoted I in Fig. 5b inset). The crystallite size of the ice calculated using the Scherrer equation, using LaB_6_ NIST 660b as a standard, gives an estimated average size of 70 nm, clearly indicating that ice formation is taking place out of the ZIF-8 cavities (inner cavities are *ca.* 1.2 nm), in close agreement with adsorption measurements described above. Afterwards, the temperature was raised again to room temperature to melt all the ice formed and later 5 MPa of methane were introduced into the capillary cell while the sample remained at 5 °C. As can be observed from Fig. 5c the synchrotron XRPD spectrum of the wet sample upon exposure to high-pressure methane perfectly fits that of the parent MOF, in close agreement with neutron scattering experiments. These results further confirm the absence of large structural deformations in ZIF-8 upon exposure to high-pressure methane, as opposed to nitrogen at atmospheric pressure.^18^

Once at high pressure (5 MPa), the reaction cell was cooled down to 2 °C and left at this temperature for 5 h before recording the SXRPD spectra. Interestingly, after the induction period under high pressure and low temperature conditions, the SXRPD profile of the wet ZIF-8 clearly shows the appearance of new peaks not overlapping with the parent MOF signals at 2*Θ*: 7.1, 7.3, 7.6, 8.4, 8.7 and 9.4° (see inset in Fig. 5 and lines denoted h, wavelength 0.4243 Å), these peaks being unambiguously attributed to the sI crystal structure of methane hydrate. The crystallite size of the hydrate calculated using the Scherrer equation is 60 nm, close to the value observed for the ice phase (lattice parameter for the methane hydrate crystal 11.9484(3) Å). It is important to highlight that under these conditions, no peaks corresponding to ice are observed, thus suggesting that all water present has been involved in the methane hydrate formation process. This observation must be attributed to the excellent water dispersion, thus being easily accessible for methane. This finding is extremely important from a technological point of view to avoid undesired weight from water non-participating in the methane hydrate formation process.

## Experimental

### Sample preparation

Metal–organic framework Basolite® Z1200 (ZIF-8) of *ca.* 4.9 microns was purchased from Sigma-Aldrich. MIL-100 (Fe) of *ca.* 150 nm was obtained using microwave-assisted (ETHOS One-Milestone) solvothermal synthesis. The synthesis involves a solution containing 2.43 g of FeCl_3_ and 0.84 g of trimesic acid in 30 mL of deionized water held at 140 °C for 15 min under microwave irradiation at 600 W. The reactant mixture was loaded in a Teflon-lined autoclave, sealed and placed in the microwave oven. The autoclave was heated up to 140 °C within 5 min and kept at this temperature for 15 min. After the synthesis, the sample was filtered and washed with methanol. The solid was finally dried at 150 °C overnight under air atmosphere. To make the hydrate structures, MOFs were humidified under water-supplied conditions denoted by *R*_w_, which represents the mass of water per gram of dry solid. The lower *R*_w_ values (*R*_w_ = 0.56, for MIL-100, and *R*_w_ = 0.01, for ZIF-8) were achieved by placing the dry MOFs in a closed container with 90% relative humidity (relative humidity was obtained using a water solution of 34 wt% glycerine). Larger *R*_w_ values were reached by adding drops of water directly to the sample.

### Sample characterization

Textural characterization of the MOFs was performed using gas physisorption measurements (N_2_) at cryogenic temperatures (–196 °C). Gas adsorption measurements were performed in homemade fully automated equipment designed and constructed by the Advanced Materials Group (LMA), now commercialized as N_2_GSorb-6 (Gas to Materials Technology; ; http://www.g2mtech.com). Before the experiment the samples were outgassed for 4 h at 200 °C under vacuum (10^–3^ Pa). Nitrogen adsorption data were used to evaluate the BET surface area, the micropore volume (*V*_0_) and the total pore volume. X-Ray diffraction (XRD) patterns of the different MOFs before and after the methane hydrate formation were recorded in a Bruker D8-Advanced diffractometer equipped with Göbel mirror (non-planar samples) with CuKα radiation (40 kV-40 mA). Measurements were made over a range of 5° < 2*Θ* < 65°, in 0.05° step width with a 1° min^–1^ scanning rate.

High-pressure analysis was performed using homemade fully automated manometric equipment designed and constructed by the LMA group, now commercialized as iSorbHP by Quantachrome Instruments. CH_4_ adsorption measurements in the dry and wet samples were performed at 2 °C and up to 10 MPa. Dry samples were outgassed at 200 °C for 4 h before the measurements, while the wet samples were frozen at –10 °C before the outgassing treatment to avoid any water loss.

### INS measurements

INS experiments were performed using the TOSCA spectrometer at the ISIS Neutron and Muon Pulsed Source, Rutherford Appleton Laboratory in the UK. Before the experiment, 0.9 g of MOF was pre-humidified with deuterated water up to a water/MOF ratio of *R*_w_ = 0.7. The wet sample (*ca.* 1.6 g) was wrapped in Al-foil and loaded into the high-pressure stainless steel cell supplied by ISIS. The sample cell and the stainless steel pipelines were surrounded by a resistance wire that allows good temperature control. The sample cell was attached to the end of the centre stick and was placed inside the TOSCA sample environment at a right position in order to overlap with the neutron beam. Before the analysis, the sample was kept in contact with methane gas at 2 °C for 5 h. Shortly after that the sample cell was properly cooled down to –263 °C with a closed cycle refrigerator (CCR). Finally, the reactor was impacted with the neutron beam (150 mA) at –263 °C.

### Synchrotron X-ray powder diffraction measurements (SXRPD)

SXRPD experiments were collected at the high-pressure/microdiffraction end station of the MSPD beamline at synchrotron ALBA in Spain, using a Rayonix SX165CCD 2D detector and a wavelength of 0.4243 Å. The experiments were performed in an *ad hoc* capillary reaction cell (fused silica capillary, inner diameter 247 μm, outer diameter 662 μm). Before the experiment, the D_2_O-containing MOF was placed inside the capillary connected to the methane gas cylinder (purity 3.5) *via* a pressure regulator. An Oxford Cryostream 700 was used to control the temperature of the sample. *In situ* SXRPD measurements were performed at 0 and 5 MPa and two different temperatures, –3 °C and 2 °C.

## Conclusions

High-pressure methane adsorption measurements show that pre-humidified MOFs promote artificial methane hydrate formation under mild reaction conditions (2 °C and 3–5 MPa). Whereas hydrophilic MOFs promote nucleation and growth in the inner cavities with a low water-to-hydrate ratio, hydrophobic systems do not allow water to access the inner porosity, thus promoting hydrate formation in the interparticle space and/or in the external surface area with a high yield. Inelastic neutron scattering experiments and synchrotron X-ray powder diffraction measurements show the first experimental evidence about the formation of methane hydrate with a sI structure on these systems. The possibility to control the nucleation process (extent of the hydrate formation, nature of the hydrate (confined or non-confined), growth kinetics, *etc.*) depending on (i) the parent MOF, (ii) the surface chemistry, (iii) the pre-humidification conditions and (iv) the reaction conditions, paves the way towards the future application of MOFs in the field of artificial gas hydrates for demanding industrial applications such as gas storage or large-distance gas transportation.

## Supplementary Material

Supplementary information
