# Supplementary material for: Paving the way for methane hydrate formation on metal–organic frameworks (MOFs)
Source: Chem Sci. 2016 Feb 19;7(6):3658–66. doi: 10.1039/c6sc00272b (PMC6008709; doi:10.1039/c6sc00272b)
Supplement: Supplementary file 1 [file SC-007-C6SC00272B-s001.pdf]

Electronic Supplementary Material (ESI) for Chemical Science.
This journal is © The Royal Society of Chemistry 2016

Paving the way for methane hydrate formation on metal-organic frameworks (MOFs)

Mirian E. Casco,[a] Fernando Rey,[b] José L. Jordá,[b] Svemir Rudić,[c] François Fauth,[c] Manuel Martínez-Escandell, [a] Francisco Rodríguez-Reinoso, [a] Enrique V. Ramos-Fernández, [a] Joaquín Silvestre-Albero [a,*]

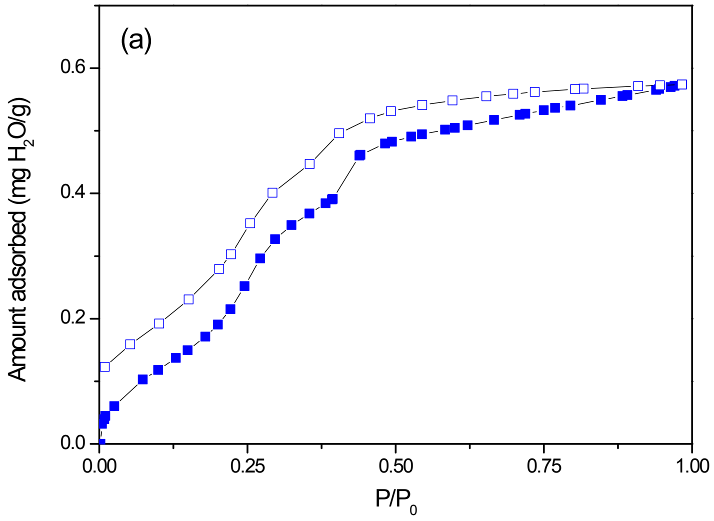

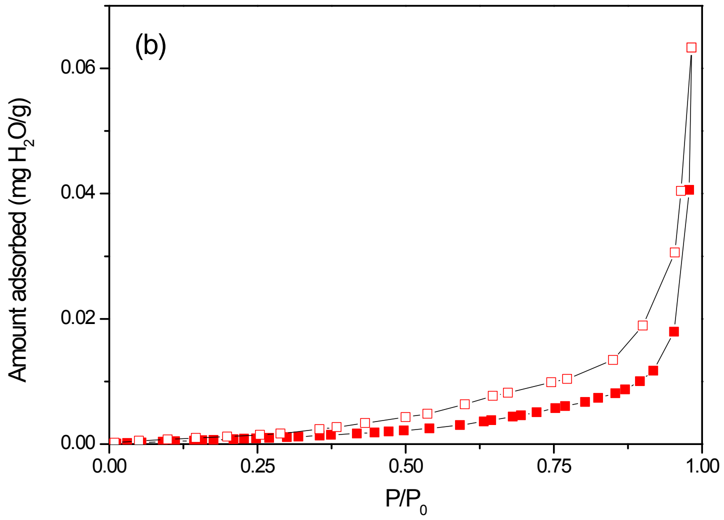

Figure S1. H_2O adsorption (full symbols)/desorption (empty symbols) isotherms at 25ºC for (a) MIL-100 (Fe) and (b) ZIF-8.

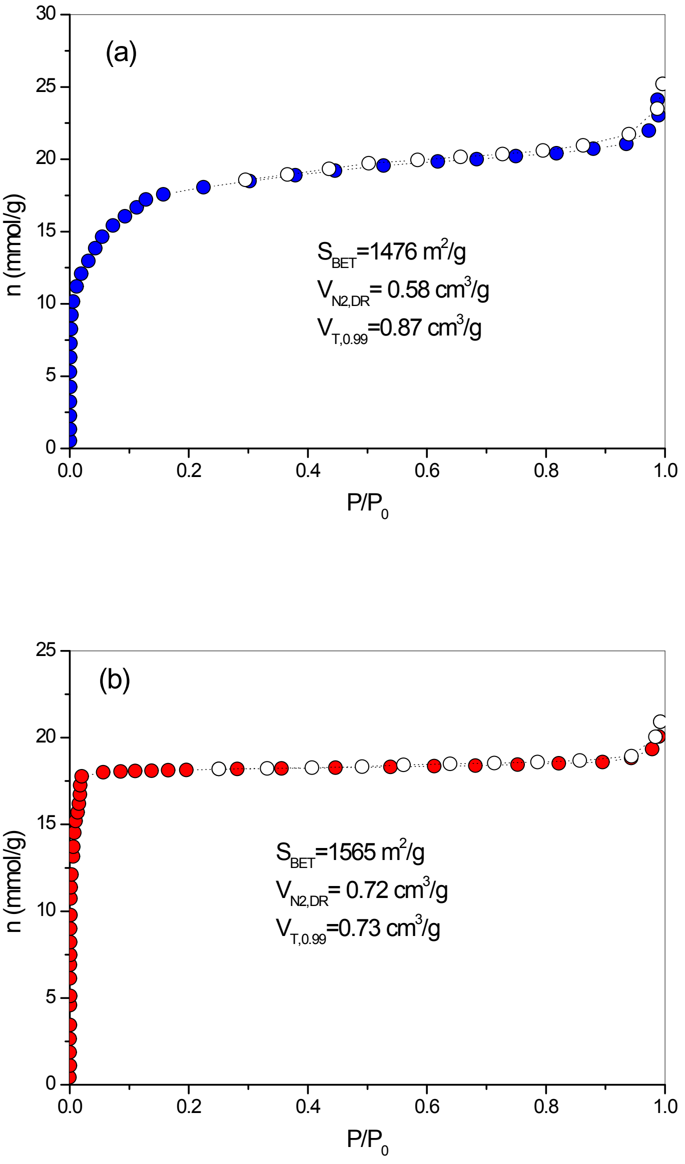

Figure S2. N_2 adsorption (full symbols)/desorption (empty symbols) isotherms at -196ºC for (a) MIL-100 (Fe) and (b) ZIF-8. Textural parameters calculated using the BET (S_{BET}) and the Dubinin-Radushkevitch (V_{DR}) equations are included.

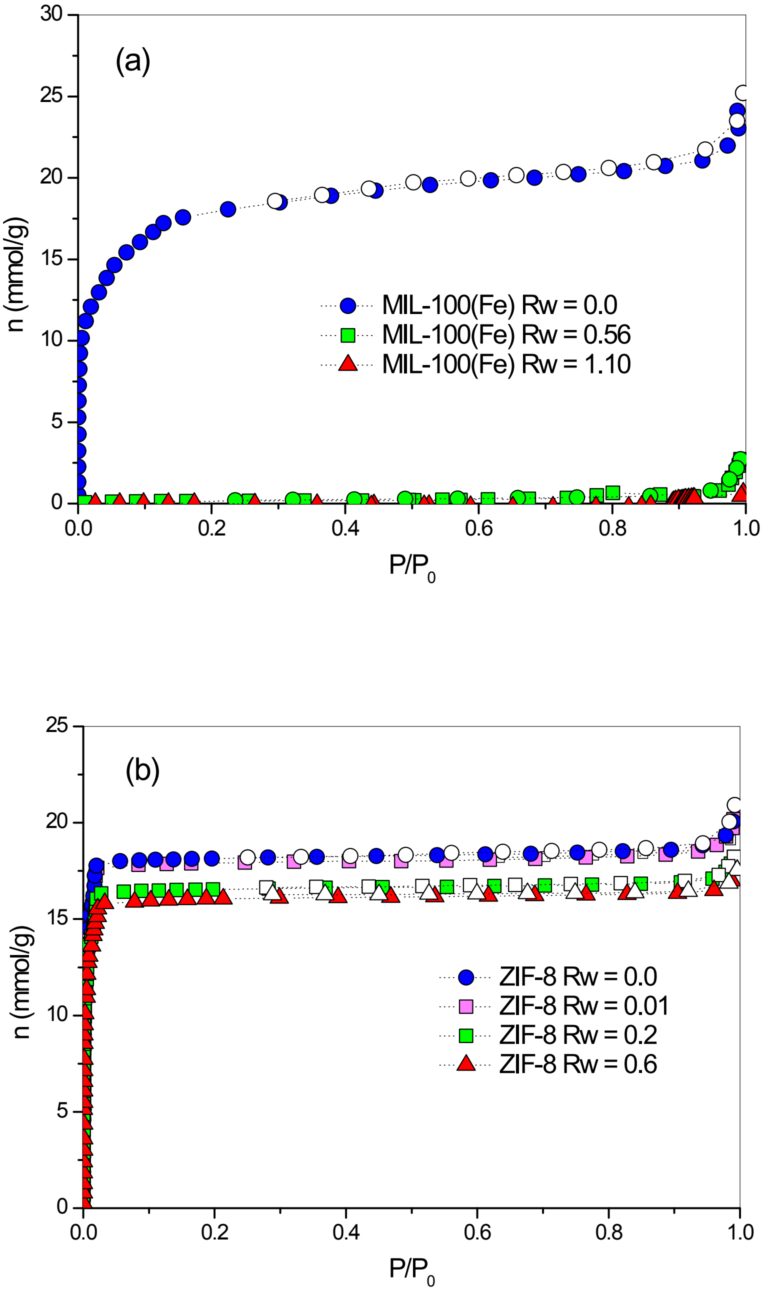

Figure S3. N_2 adsorption (full symbols)/desorption (empty symbols) isotherms at -196ºC for (a) MIL-100 (Fe) and (b) ZIF-8, before and after water pre-humidification.

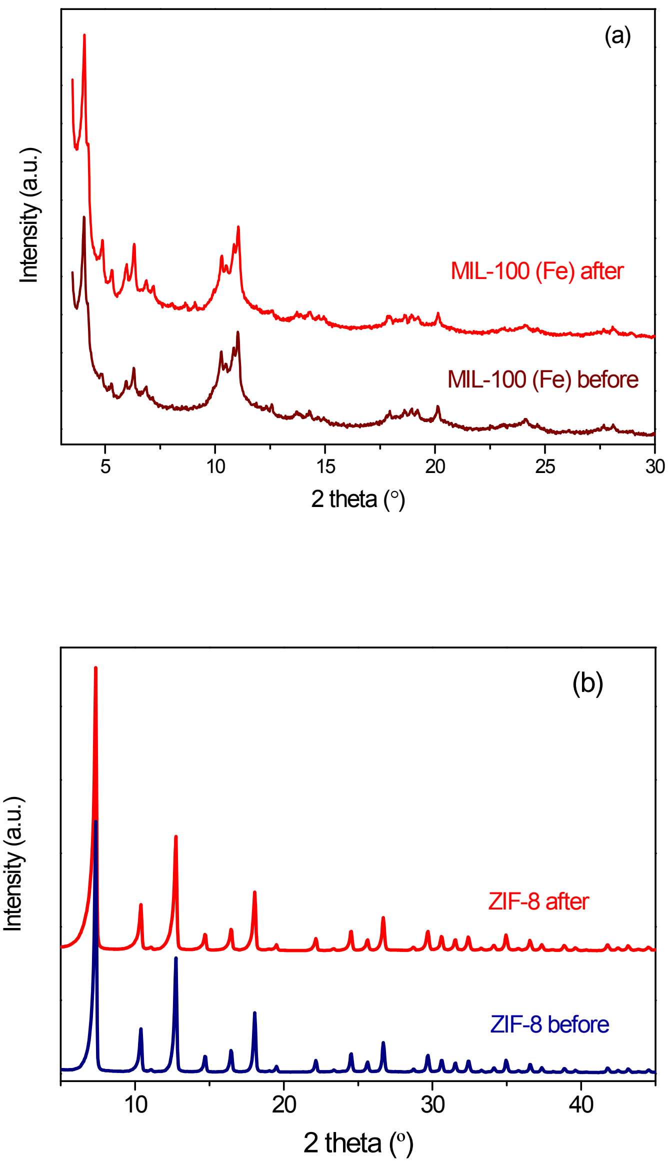

Figure S4. XRD pattern for (a) MIL-100(Fe) and (b) ZIF-8 before and after the formation of the methane hydrate.

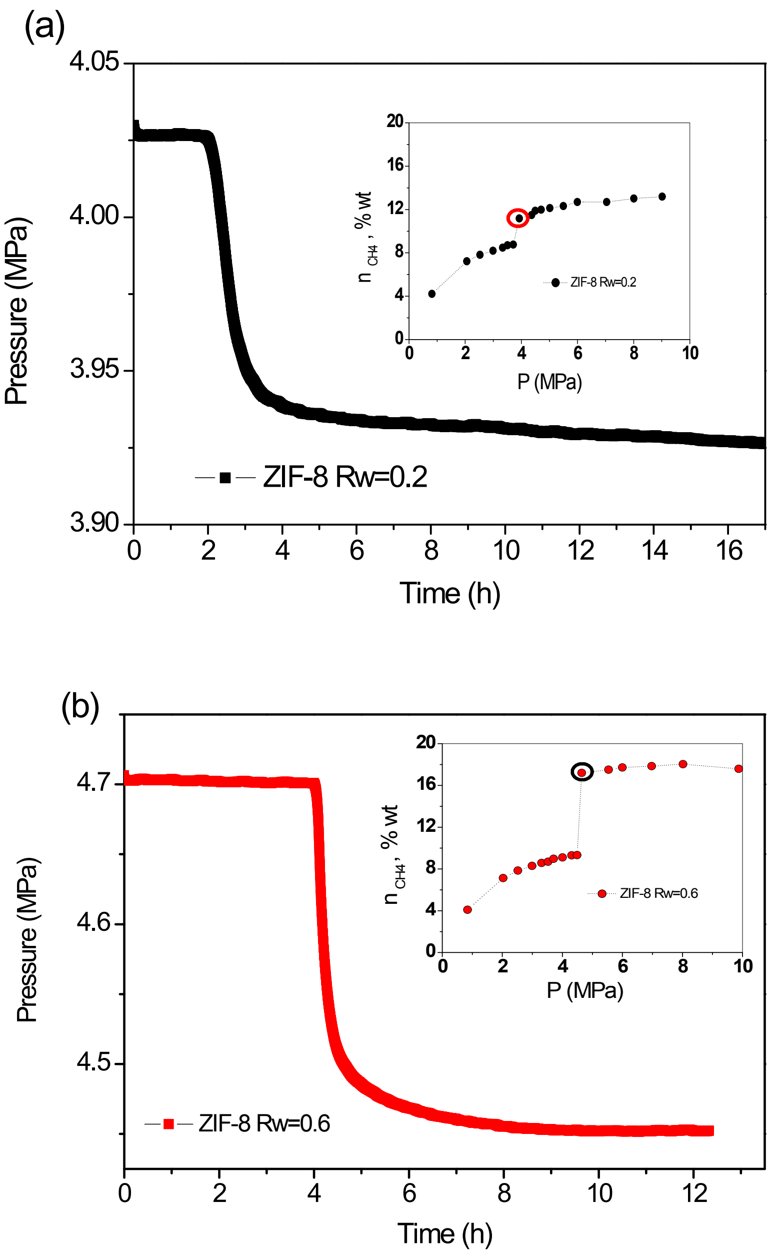

Figure S5. Adsorption kinetics for ZIF-8 after pre-humidification with (a) Rw=0.2 g/g and (b) Rw= 0.6 g/g, at the point of the adsorption isotherm associated with the jump (see inset).

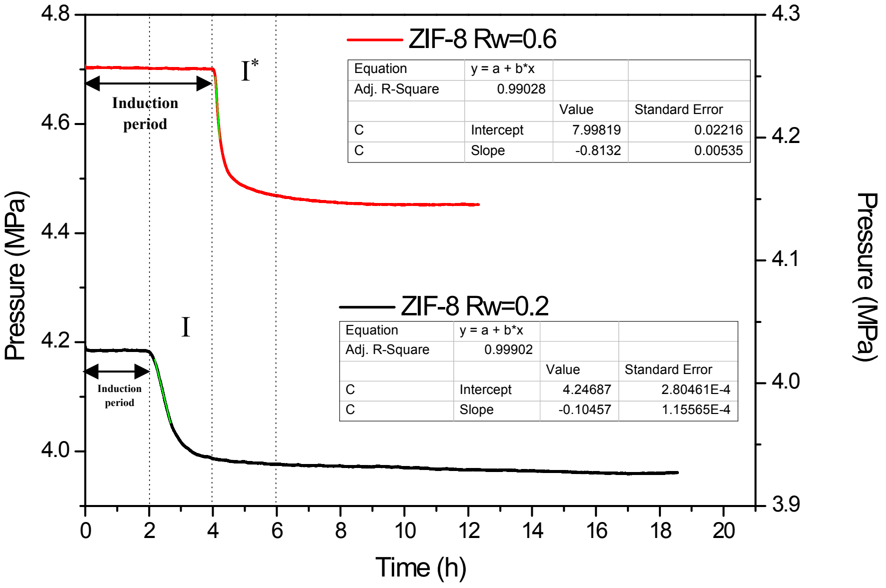

Figure S6. Comparative of the adsorption kinetics for ZIF-8 after pre-humidification with (a) Rw=0.2 g/g and (b) Rw= 0.6 g/g, at the point of the adsorption isotherm associated with the jump. The slope of the crystal growth zone (I and I*) is included in the inset table.
